# Decision-making criteria for medicine reimbursement in Slovenia: an expert panel discussion

**DOI:** 10.1186/s12913-018-3299-z

**Published:** 2018-06-27

**Authors:** Andreja Detiček, Andrej Janzic, Igor Locatelli, Mitja Kos

**Affiliations:** Chair of Social Pharmacy, Faculty of Pharmacy, University of Ljubljana, Askerceva 7, 1000 Ljubljana, Slovenia

**Keywords:** Decision-making criteria, Medicine reimbursement, Focus group, Health technology assessment

## Abstract

**Background:**

Rational and transparent Health Technology Assessment and reimbursement decision-making are crucial for healthcare system sustainability. A part of the reimbursement process are decision-making criteria which should be clearly defined.

**Methods:**

The study aimed to obtain an insight into understanding and relevance of potential criteria for the medicine reimbursement decision-making process in Slovenia.

A semi-structured guided focus panel was performed in June 2017 with five Slovenian experts covering principal healthcare system sectors. First, criteria understanding and relevance for medicine reimbursement decision-making were discussed. Second, healthcare priorities and societal values affecting decision-making were debated. The analysis was carried out with NVivo 11 by two independent researchers who coded the verbatim transcript in three coding steps based on the experts’ interpretations and original ideas.

**Results:**

Seven decision-making criteria were derived. Among those, the impact a disease has on the lives of patient family and caregivers and the indirect medicine benefit for them were new aspects comparing to the existing criteria set in Slovenia. The experts expressed that the same decision-making criteria are relevant for evaluating any health technology, allowing for different criteria weights. They also suggested a system that would allow re-evaluation of reimbursement decisions once real-world clinical data are available.

**Conclusions:**

As proposed by the international frameworks and tools, the Slovenian healthcare experts consider including multiple aspects more ethical and comprehensive than considering a single criterion, e.g. cost-effectiveness, existing in some healthcare systems. They recognize that in the existing decision-making process, health perspectives of the public represent a largely missed aspect.

## Background

The Slovenian healthcare system is a Bismarck-type social insurance system. Compulsory health insurance contributions gather in a common fund. Private funds also exist as complementary health insurance paid by 90% of the population. However, providing healthcare is mostly in public domain [1, 2].

The compulsory insurance fund is managed by the Health Insurance Institute of Slovenia (HIIS), an autonomous national healthcare decision-maker and payer who also makes decisions about medicine reimbursement. The reimbursement of other health technologies is decided by the Health Council at the Ministry of Health [1, 2].

The processes of deciding on financing new technologies, including the selection of criteria, were set by each institution separately. Therefore, the set of criteria differs between institutions. However, some of them, such as therapeutic relevance, level of evidence and concerned population, are common and many others are similar to the criteria used in other countries [1–4]. However, their definition allows different understanding and interpretation, such as the criterion “public health relevance”, usually only considered as great, medium or small [3]. Also, some criteria are defined very narrowly, e.g. the criterion “ethical aspect” only including two options – whether the medicine treats a very severe or a rare disease or not [3]. Therefore, the meaning of criteria is not necessarily completely clear and understood in the same way by the evaluators, what prevents comparability and transparency of reimbursement decisions. Additionally, since the core criteria were set more than a decade ago, some potentially relevant aspects to decision-making might be left out.

In medicines reimbursement, the HIIS makes decisions according to the criteria and negotiations with pharmaceutical companies. So far, the HIIS was able to reimburse a large number of important breakthrough innovative medicines, mostly with prescribing restrictions for patients who are most affected by the disease (e.g. patients who do not respond to any other available therapies) [5, 6]. On the other hand, strict prescription restrictions mean that there might be a larger pool of patients who would probably benefit of new medicines according to their indications. Despite the restrictions and the fact that the introduction of cost-containment measures helped decrease the expenses on other medicines, e.g. the therapeutic reference pricing system introduced in 2013, medicine expenses are increasing. This is particularly due to so called “expensive medicines”, including mostly new biological medicines and other expensive medicines exceeding a cost of two thousand euros per patient per year, which increased by 5.3% between 2016 and 2017, and represented 30.8% of the total medicine expenditure by the HIIS [5]. Additionally, new pricy medicines are regularly introduced on the top of the current treatments presenting further pressure on budget for medicines, which will have to be wisely managed with very clear criteria of which medicine are reimbursed.

As healthcare reimbursement decision-making continuously evolves, many tools using multiple criteria decision analysis approach were developed worldwide to help making decisions [7–19]. Different criteria frameworks were proposed, e.g. the European Network for Health Technology Assessment framework (EUnetHTA) [11, 20–25]. They suggest considering multiple aspects also concerning fairness, solidarity, patient and community perspectives, and healthcare system organization [20–25]. They state criteria content should be well defined and understood by all stakeholders and used either intuitively or quantified by weights [10, 12, 25–27]. Following these guidelines, the Slovenian medicine reimbursement process and criteria need to be reconsidered to optimize decision-making. Assuring good criteria understanding and their significance for the local healthcare system is fundamental [10, 12].

Therefore, we aimed to describe the understanding of different criteria potentially used in the Slovenian medicine reimbursement decision-making and their relevance among key healthcare experts.

## Methods

### Study design

A semi-structured focus panel with five Slovenian healthcare experts was performed in June 2017. The 2-h discussion was held in an academic environment and was audio-recorded in total upon written consent and transformed into a verbatim transcript. Further, two coders (AD, AJ) carried out theme analysis with NVivo 11 in three coding steps.

### Expert panel selection

Participant selection was purposive so that principal healthcare system sectors were covered. Five experts participated:

- a decision-maker with over 20 years’ experience in medicine reimbursement decision-making and currently involved in medicine reimbursement at the HIIS, actively involved in health policy-making regarding medicine reimbursement (a doctor);
- a clinical expert in infectiology specialised in epidemiology and clinical pharmacology, working at the leading clinical institution in the country with experience in resource allocation, and also a former member of the Health Council at the Ministry of Health, responsible for the preparation of scientific guidelines and standard operating procedures of the use of new health technologies including medicines (a doctor);
- a clinical expert in cardiology managing a division at the leading clinical institution in the country, an important representative of the national cardiologist association involved in preparation of treatment guidelines, also experienced in healthcare resource allocation, and a former member of the Health Council at the Ministry of Health, involved in health technology implementation into clinical environment (a doctor);
- a regulatory and pharmacoeconomics expert with more than 15 years’ experience at the public Agency for Medicinal Products and Medicinal Devices of the Republic of Slovenia dealing with health technology assessment and medicine prices and actively involved into policy-making regarding medicines on national and international (the European Union) level, and also a member of the Slovenian coordination about Health Technology Assessment collaboration in the European Union (a pharmacist); and
- a public health expert at the National Institute of Public Health with several years’ experience in research and implementation of quality in healthcare and evidence-based healthcare policy-making at the Ministry of Health (a doctor).

The participants were familiar with the research group due to previous research collaborations and professional encounters. They were invited to participate via e-mail where the purpose and the context of the study were presented. They expressed the willingness to participate for recognizing the challenge of medicine reimbursement decision-making in view of rising patient needs and limited resources.

### Criteria domains and themes discussed

Criteria domains and their brief description were gathered from various sources; the Slovenian criteria for reimbursement of medicines, the Slovenian criteria for evaluation of other health technologies, the all-European core set for pharmaceuticals developed by the European Network for Health Technology Assessment (EUnetHTA), and an extensive literature review including worldwide criteria [3, 4, 20, 22]. The complete set used to lead the discussion consisted of nine domains is presented in Table 1.

**Table 1:** Decision-making criteria domains used in the guided discussion with the expert panel

Domain	Domain content and attributes	Sources†
1 Disease impact, Health problem	Disease severity; disease determinants; disease burden; current management of the condition; quality of care; public health interest	EUnetHTA, EVIDEM framework, Guindo et al. 2012, Slovenian criteria medicines and other health technologies
2 Population and healthcare priorities	Target population; population effect; individual effect; size of population affected by disease; vulnerable and needy populations; public health interest; mission and mandate of the healthcare system	EUnetHTA, EVIDEM framework, Guindo et al. 2012, Slovenian criteria medicines and other health technologies
3 Ethical aspect and social aspect	Priorities, respect for persons, justice, ethics, equity, solidarity; patient autonomy; legislation; access to medicines; ethical consequences of health technology assessment; patient and social group perspectives; population priorities; communicational aspects; need for the treatment; patient initiatives	EUnetHTA, Guindo et al. 2012, Slovenian criteria for medicines
4 Medicine description, characteristics and therapeutic context	Type of medical service; features of the medicine and its regulatory status; investments, tools, training and information required to use the technology; treatment alternatives; need for the treatment; clinical guidelines and practices; pre-existing use of the medicine	EUnetHTA, EVIDEM framework, Guindo et al. 2012, Slovenian criteria medicines and other health technologies
5 Efficacy and Health Benefit	Clinical effectiveness; mortality and morbidity; health-related quality of life; benefit-harm balance; patient-reported outcomes; improvement of patient reported outcomes, convenience and adherence	EUnetHTA, EVIDEM framework, Guindo et al. 2012, Slovenian criteria medicines and other health technologies
6 Safety	patient safety; occupational safety; improvement of safety and tolerability; safety risk management	EUnetHTA, EVIDEM framework, Guindo et al. 2012, Slovenian criteria medicines and other health technologies
7 Relevance of evidence	Quality of evidence (research ethics, completeness and consistency of reporting, validity); evidence uncertainty (available evidence and the power of evidence)	EVIDEM framework, Guindo et al. 2012, Slovenian criteria medicines and other health technologies
8 Economic aspect	Costs and economic evaluation of the medicine (CEA, CUA, BIA); characterizing data uncertainty and heterogeneity; validity of economic models; opportunity costs; value of a medicine	EUnetHTA, EVIDEM framework, Guindo et al. 2012, Slovenian criteria medicines and other health technologies
9 Organizational and legal aspects, medicine implementation	Organizational requirements and capacity to implement; healthcare system structure; management; barriers and acceptability; medicine flexibility, integration and system efficiency; patient autonomy and privacy; equity in healthcare; authorization and safety; ownership and liability; market regulation	EUnetHTA, Guindo et al. 2012, Slovenian criteria medicines and other health technologies

Legend: † EUnetHTA - European network for Health Technology Assessment [20]; EVIDEM framework - framework Evidence in Decision-Making [21]; Slovenian criteria set for reimbursement decision-making of medicines [3]; Slovenian criteria set for the assessment of other health technologies [4]; Guindo et al. 2012 [22]

The experts debated about the main topics concerning reimbursement decision-making process for medicines in Slovenia:

- in-depth understanding of the existing criteria and nine criteria domains originating from the literature review and their relevance for local decision-making [2, 3, 20–22],

- the significance of particular patient/disease groups potentially considered healthcare priorities that could be exposed in the evaluation through reimbursement criteria.

At the end of the discussion, all participants were given the chance to expose any additional issues they felt were not yet mentioned.

### Research group

The research group consisted of four researches, all working in academia in the field of Health Technology Assessment, including comparative effectiveness, pharmacoeconomics, and evaluation of performance of medicines in real-life settings; two senior researchers and faculty teachers, a researcher in the field of health technology assessment, and a third-year doctoral student. Researchers were familiar with qualitative research and had already performed qualitative studies, including focus groups.

The facilitator (AD) performed the literature search, was involved in the preparation of questions and was trained to lead the discussion by senior researchers (MK, IL). The discussion was observed by two other researches (AJ, IL) that were both making notes about non-verbal signs. The facilitator (AD) and the main observer (AJ) were then involved in data analysis and themes derivation as data coders.

### Qualitative data analysis

Two coders (AD, AJ) derived all themes from participants’ interpretations and ideas using the verbatim transcript, including non-verbal expressions detected by both discussion observers.

They coded themes in three steps. First coding was independent using the key phrases mentioned at least once, e.g. ‘relative effectiveness’. Then, the coders transformed their coding trees into one upon agreement and determined new theme hierarchy, forming the proposition of a set of reimbursement criteria. Then, when necessary adjustments of coding trees were made in agreement to obtain the final themes, that was confirmed by both coders. They used NVivo 11 for transcript analysis and built a hierarchy chart based on the number of citations under each topic. Thereupon, the coders prepared a suggestion of revised decision-making criteria. The final set, the transcript of the discussion, and the findings of the study were not reported to participants for additional feedback.

The researchers followed the Consolidated criteria for reporting qualitative research (COREQ) [28, 29].

## Results

**Fig. 1:**
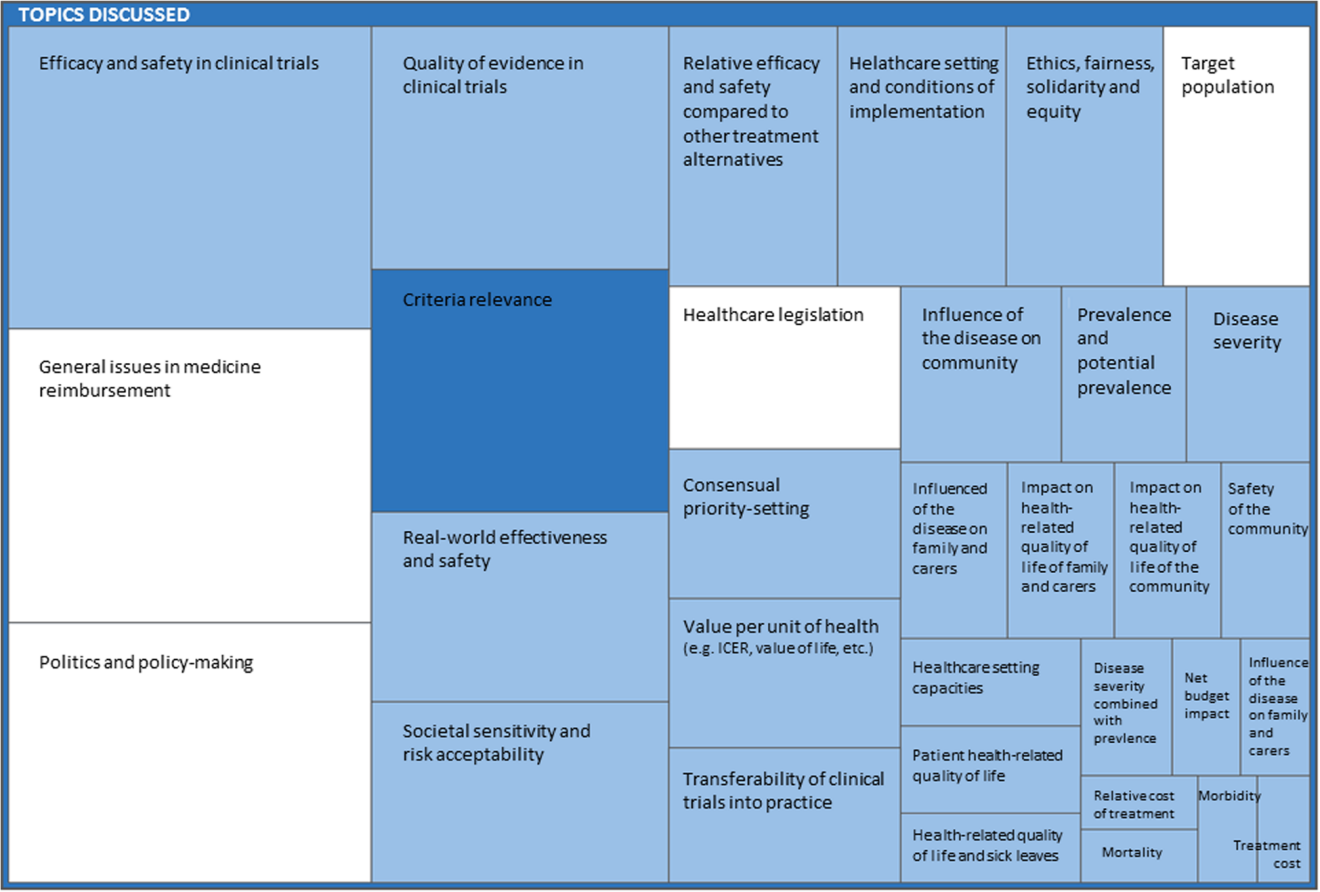
Hierarchy chart presenting all discussed topics about criteria understanding and relevance. Legend: Rectangle size represents how heavily the topic was discussed according to the number of citations. Light blue – the content of decision-making criteria; dark blue – criteria relevance; white – supportive themes

The subtopics generating the content of the proposed criteria and other topics discussed are presented in a hierarchy chart (Fig. 1).

### Criteria derived from the discussion

**Fig. 2:**
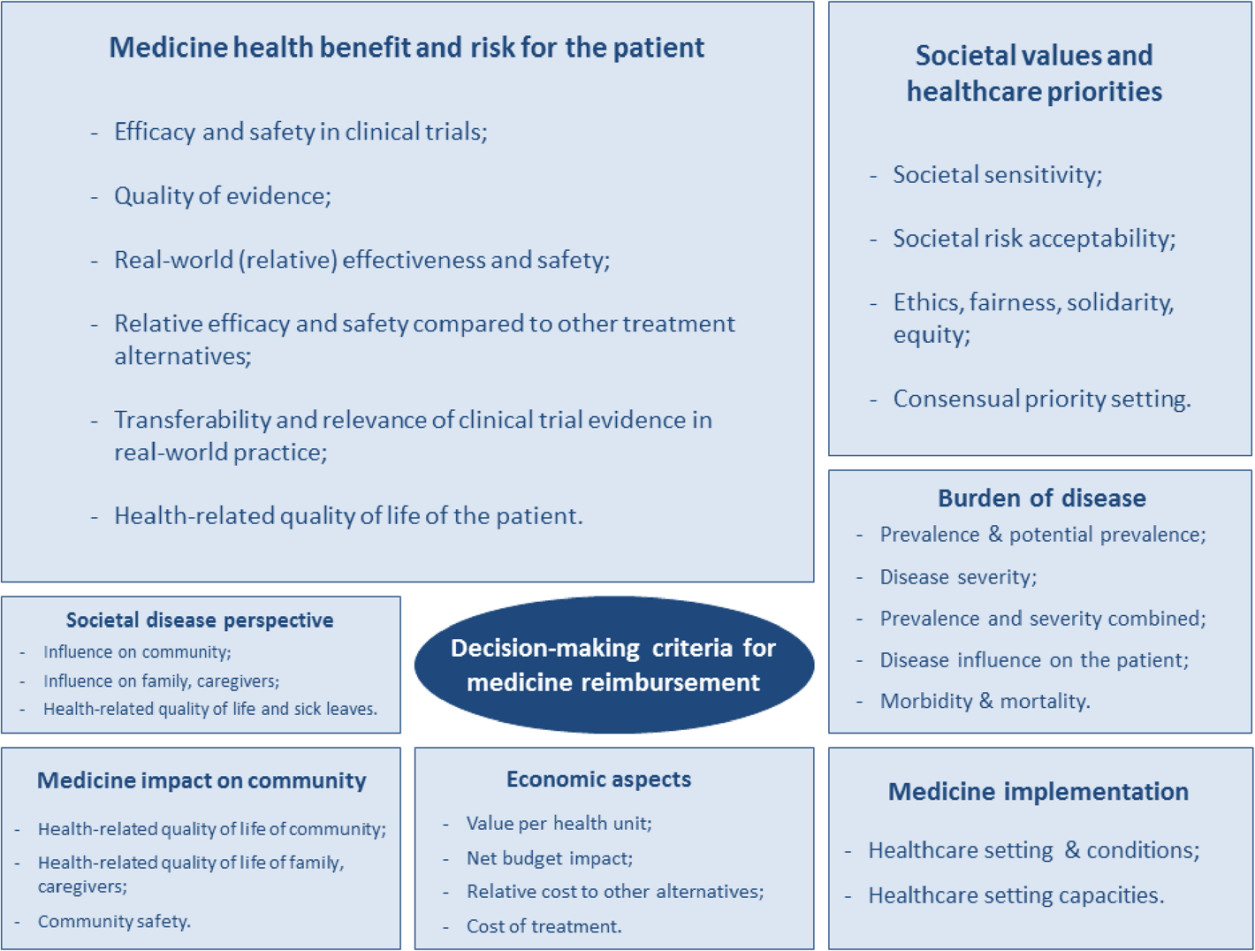
Seven criteria relevant for medicines reimbursement decision-making derived from the focus panel. Legend: The rectangle sizes show how heavily the topics were discussed upon according to the number of citations under each topic

The researchers derived seven criteria considered for medicine reimbursement decision-making from the discussion. Figure 2 presents these criteria and their main elements.

#### Medicine health benefit and risk for the patient

General experts’ view was that above all, a new medicine should be proven effective and safe with quality designed and performed clinical trials with transferable results to the real clinical practice. The importance of effectiveness as criterion was emphasised several times. One clinician expressed:

“*There is no point in talking about e.g. disease prevalence or disease severity if the medicine is not effective; because even if the disease it is supposed to treat is very prevalent and very severe, the effect of the medicine would be very small.*”

In addition to that, the regulatory expert exposed that effectiveness should also be considered in real clinical settings:

“*there* are *more “types” of effectiveness: first there is the efficacy leading towards medicine authorisation, then there is effectiveness, expressed in the real circumstances… it’s important to understand “effectiveness” on both levels.*”

However, all experts agreed that medicine’s relative effectiveness is the most relevant aspect of medicine health benefit. The clinician specialised in epidemiology and clinical pharmacology elaborated:

“*it’s about medicine effectiveness in comparison to a similar medicine; if a medicine is more effective than another, that is why we need it, that is why we need to use and finance it… we’re looking for the health benefit we don’t yet have in another medicine.*”

The medicine reimbursement expert added:

“*Medicine’s added value or relative effectiveness is of extreme importance; it’s the effectiveness versus placebo and versus active comparator which should be optimally chosen.”*

Along discussing the importance of medicine effectiveness, the panel saw safety tightly connected with efficacy as the assessment of risks and benefits is usually done simultaneously:

“*safety goes hand-in-hand with efficacy; if a medicine has a poor risk-benefit balance, it is not even authorised for the market, so these two always have to go together… then, there are mechanisms to maintain or improve safety and to know more about it, such as post-authorisation safety studies*”, as the regulatory expert explained.

One of the clinicians partially agreed that medicine safety is crucial but disagreed that this should be as important in decision-making as effectiveness.

Another clinician advocated the importance of safety aspect and concluded the debate with the words:

“*There is such a fine line between the importance of medicine benefit and the importance of its hazard.”*

When asked about what measures or aspects are considered under safety, the experts unanimously replied that these are medicines’ adverse events and the medicine reimbursement decision-maker explained that mostly individual patient safety is in question when talking about medicine safety.

However, experts spontaneously offered several opinions on the quality of evidence and the lack of comparisons of different treatments. One clinician pointed out that:

*“there are very few head-to-head comparisons… Data quality is crucial. I could hardly say that it is separated from benefit and safety. Nevertheless, this is what the use of a medicine is founded on.”*

Further, the panellists presented the concept of personalized medicine and the transferability of results from clinical trials into practice as the most challenging issues for decision -makers. The medicine reimbursement decision-maker argued:

*“… (in clinical trials) there are two options which comparator to choose: either the most commonly prescribed medicine in practice or the most suitable medicine recommended by the guidelines. But there could be a huge difference between both comparisons and that’s the most common problem in reimbursement decision-making – the choice of comparator is sometimes biased or, for example, the dosage of comparator is too low…”.*

The public health expert added:

*“As a “criterion”, quality of evidence should be taken into account at the same time when we consider efficacy and safety. We have to keep the question “how sure are we?” in mind… what is the reliability of these data”.*

A clinician explained:

*“Some studies use small number of patients, some have inadequate comparisons, then there are studies that don’t consider certain indications… so, we don’t actually have enough information to decide according to the criterion we consider the most relevant!”.*

*“Medicine reimbursement decisions are also based on recommendations (by the guidelines) and the level of evidence is considered*”, concluded the cardiologist, also involved in guideline preparation.

Further, all experts agreed that real-life data on effectiveness and safety should both be considered in decision-making when they are available. The expert explained:

“*the real-world data are becoming a paradigm; target patient populations are smaller and the information of real-world relative effectiveness has become more important. Patient registries are also the basis for showing medicine health benefit… real-life data are also important evidence.*”

Finally, patient’s health-related quality of life was also perceived as medicine health benefit as the public health expert elaborated:

“*We need to use measures of health benefit that exceed the basic evidence of medicine’s efficacy but concern the quality of life. But how are we going to come to an agreement about one medicine being 10 or 20 or 50% better than another? Is QALY going to be the measure?*”

The other experts unanimously agreed but pointed out the concerns about appropriate comparators in clinical trials once more.

#### Societal values and healthcare priorities

The whole panel was certain that priorities should be based on population values and formed on general population ethics, fairness, solidarity, sensitivity for perceiving a disease as needed to be treated and risk acceptability. One stated:

*“Ethics is the compassion for those who need help from the community and whom we need to provide a decent life”.*

They illustrated that sensitivity of the community for its weaker members is an important aspect behind priority setting:

- “*There are certain health problems that are not so common but are so severe that the community understands them as needed to be resolved, like some rare diseases or Haemophilus B vaccination that we reimburse…”* was the clinician’s opinion meeting patients daily;
- “*It’s about the attitude that an individual has towards himself, their own health and the health or sickness of others*” added the regulatory expert.

Further, an expert perceived risk acceptability as an aspect of population sensitivity differing between communities in how much one is willing to bear:

“*Some societies are willing to accept more adverse events than others… the perception* of *patients (about medicine health benefit and risks) affects medicine use.”*

From the regulatory point of view, the expert added:

“*Societal values are very important and affect the priorities and represent the link to legislation… sooner or later the regulations about medicine reimbursement, price-setting etc… reflect these values.”*

However, they all shared the opinion that priority-setting should not be in domain of decision-makers, but the healthcare priorities should be defined in advance by the general public who contributes to the national healthcare fund and only then considered in reimbursement decision-making as it was explained:

“*Of course, one of the criteria affecting decision-making should be what the healthcare priorities are… but healthcare priority-setting is a part of another - a healthcare policy-making - discussion*”.

Another expert said:

“*The question is: what the representative opinion of our people is?”*

Finally, all experts agreed that decision-makers should consider healthcare priorities through reimbursement criteria whichever these are (e.g. as rareness of a disease, severe debilitating diseases or particular patient populations considered priorities by the general public, such as the children etc.). However, these should be set separately by broader public debate and then used further in both: forming the health policy and in medicine reimbursement decisions.

Moreover, the public health expert’s opinion was that all of the aforementioned concepts supposedly have very much in common with other societal criteria:

“*It about enabling equity… Ethical and social aspects come together when answering to our inner sense for justice: it is unjustly that someone with a rare inborn disease is left alone to himself, and it is unjustly that someone living in certain social circumstances has no rights”*.

#### Burden of disease

The panel defined burden of disease as prevalence, potential prevalence (a risk for disease outburst), morbidity, mortality, and severity of disease summing up in the impact of disease on an individual. The epidemiology-oriented infectiologist described:

*“Disease impact and health problem are represented by disease severity, which means how a disease affects health… First, how a disease affects the patient in terms of morbidity and mortality, then, population-wise, it’s the prevalence. Both dimensions – whichever increases, it increases the health problem”.*

However, an expert exposed the public health perspective:

*“The prevalence could be the measure, but in a public health view, we are also worried about the potential prevalence of a disease, e.g. in infectious diseases”.*

The thought was continued by another expert:

*“Especially, if a certain health problem is poorly covered by the existing standard of treatment, its impact becomes larger… so, two aspects are important: quantitative epidemiological aspect and how a patient and its environment are affected”.*

Altogether, a combination of these factors should be considered in medicine reimbursement decision-making as one of the clinicians concluded:

“*Shouldn’t we combine prevalence with disease severity? If something is very common but has little effect, well… It has to be a suitable combination of both”*.

#### Societal perspective of the disease

The experts believe it should be considered that a disease does not only affect the patient but also their relatives, caregivers and the whole community. One expert expressed:

*“We need to consider what are the social consequences and aspects of the fact that some health problem exists*”.

The medicine reimbursement decision-maker mentioned sick leaves and health-related quality of life of all affected by the disease:

*“I would put sick leaves and QALYs (quality-adjusted life years) and DALYs (disability-adjusted life years), the quality of life in this aspect. I see it at the population level”.*

“*…this is a social aspect in the sense of how a disease affects the whole community”,* continued the infectiologist and the regulatory expert agreed:

“*Exactly. Not only the effect a disease has on the patient but to all of those who are dragged behind.”*

The experts also commented that it is our responsibility to include such aspects in decision-making, as was pointed out by one expert:

“*Social aspect should be considered because of our demographic image”*, pointing at the prevailing elderly population.

One of the clinicians shared the opinion and added that this is the aspect of “social responsibility”.

The panel saw disease impact on society connected with medicine impact on society and perceived both as currently missing aspects, as the medicine reimbursement decision-maker elaborated:

“*These aspects are what we are missing now or at least we ignore them.*”

#### Economic aspects

The panel discussed treatment cost, net budget impact and willingness to pay as economic aspects needed to be considered and very important in reimbursement decision-making. The regulatory expert with pharmacoeconomics background pointed out that we need to be realistic about the affordability in Slovenia – that it is a small pharmaceutical market and that its purchasing power is lower than in many other European countries. Then, the expert defined cost as:

“*The basic equation is simple:* cost *is price multiplied by volume.”*

From the clinician point of view, the cost could include more:

“*It is an absolutely broader concept than medicine price only. It is the cost of treatment containing, for example sick leaves for taking care of a patient with Alzheimer’s disease, and diagnostics”.*

The public health expert presented a dilemma of which perspective (societal or healthcare payer) to use in pharmacoeconomic analyses and stated that budget impact is a separate aspect than medicine cost or cost-effectiveness:

“*We should defend the societal perspective. But it is complex to calculate and it does not reflect the relevant information for the payer. I believe there is a general intrinsic contradiction (in performing pharmacoeconomic analyses) … Also, I would say that budget impact is a separate aspect than cost-effectiveness… both aspects should be used as separate aspects and including all (medical) costs, related to medicine use”.*

Further, the panel strongly discussed about the value per unit of health rather than cost-effectiveness after a clinician arose the question:

“*We are talking about the medicine and the models… but, within the economic aspect, do we know what value to attribute to life?”*

Three experts mentioned that in the previous years such a value was set for reimbursement decisions within the Health Council for other health technologies and set at 3-times gross domestic product which was used according to the examples in other countries. They concluded that it was used in reimbursement decisions, and a former member of the Health Council added:

- *“A threshold or a value of life is some kind of a ‘soft’ value affected by other aspects we have already talked about previously. But the economic aspect is important comparatively.*”

The other experts agreed that a relative comparison of costs or cost-effectiveness between different alternatives is crucial.

Finally, basing on the experience in healthcare management and reimbursement decision-making at the Health Council, an expert replied that it would be nice to have the thresholds set, besides the cost-effectiveness threshold, and emphasised the importance of the societal consensus about it.

#### Medicine implementation

Initially, the general opinion of the panel was that how healthcare is organized is not something that should necessarily be considered in medicine reimbursement decision-making, since this is a domain of healthcare policy-making and not decision-making. However, the expert facing reimbursement decisions regularly expressed the importance of healthcare legislation and organisation for reimbursement decisions:

“*The background needs to be assured so you can correctly assess medicines… but it is not a criterion on its own, in my view.”*

The experts felt that the healthcare system needs to provide the fundamental framework for appropriate medicine use and simultaneously enable solidarity for all insured, explained by the clinician meeting patient every day:

“*Implementation of a medicine needs to be considered comprehensively; medicines are paid from public money, meaning they have to be accessible for everybody financially participating while guaranteeing solidarity… for example, some medicines are available in ambulatory care, but not in hospital care, some medicines are reimbursed in one hospital but not in another… some kind of an organizational system needs to be developed to overcome that”.*

The public health expert oriented into quality in healthcare continued:

“*In a way, it is immoral to implement a health technology if there are no capacities in the healthcare system to use it… maybe we shouldn’t reimburse a new medicine today, but first enable the capacities to face the problem.”*

Eventually, they all agreed that implementation is an important aspect for decision-making and the medicine reimbursement decision-maker concluded:

“*It is extremely important that by decision-making we improve the organization of healthcare, enable better patient flow and not worsen the situation, like increasing the number of due to putting a medicine on a certain list*”.

#### Medicine impact on community

The public health expert stressed the importance of medicine impact on quality of life of others besides the patients:

“*One of the criteria is the effect of the medicine on other people. We usually deal with the effect on the patient and how it affects their quality of life… But the relatives who have to take care for them must also be considered”.*

The disease and medicine impact on community overlap and the experts agreed these are both missed aspects in the current decision-making process. An expert responsible for allocating resources explained:

“*If an anti-dementia medicine decreases the burden of patient’s relatives, caregivers, especially if the patient lives at home, we officially do not care about it at all. Unofficially we do, of course, but this is not an official criterion now*”.

Additionally, an expert presented medicine impact on community safety as a form of societal responsibility:

*“It is not only the patient that needs to be safe but also the whole community: for example, the problem of antibiotic-resistance, the intentional misuse of certain medicines or antipsychotics where safety of medicine use is under question”.*

### Relevance of the criteria

The whole panel believed medicine reimbursement should be considered broadly according to the medicine reimbursement decision-maker’s words:

*“Our decision-making system comprises multiple aspects and I believe in this way is more ethical and comprehensive”.*

The whole panel also agreed on considering all aspects discussed as criteria in decision-making: the expert experienced in health technology evaluation said some aspects are more relevant than others but should all be considered in reimbursement decisions about all health technologies, not only medicines. Another expert additionally mentioned the problem of transforming these aspects into criteria and how to use them – either by allocating points or as yes/no decisions.

Regarding which aspects are more relevant than others, the experts considered *medicine health benefit and risk* as the central aspect in medicine reimbursement decision-making as emphasized by the quotation:

“*Efficacy is the crucial criterion. Everything else is built around it*”.

The experts understood safety as a complementary aspect to efficacy while the quality of evidence is what efficacy and safety stand on. However, not all experts perceived safety as relevant as effectiveness, as one clinician expressed:

“S*afety seems less relevant to me than efficacy and medicine health benefit. Why? Because it is already ‘cut off’ previously by regulatory bodies. So, at the stage of deciding about reimbursement, the medicine is already proven safe and cannot be that harmful.*”.

Reversely, the expert with less experience in clinical practice argued that safety aspect is important and could not be taken for granted in reimbursement-decision making even if proven previously by regulatory agencies and said:

“*this is the second most important question (in reimbursement decision-making)*”.

A clinician summed up what most relevant criteria should be:

“*The extremely important aspects to me are efficacy and health benefit, safety and the relevance of evidence which are essential; but, of course, we can do nothing without the economic aspect”*.

Therefore, the panel considered the economic *aspect* the second crucial criterion. One expert simply explained:

*“It’s about what we invest in and what we get”*.

The expert involved in healthcare policy-making added:

“*The economic aspect is crucial and will be more and more crucial according to the trend of developing more medicines for more narrow populations and, therefore, more expensive per unit.”*

Further, the panel understood burden *of disease* tightly related to *societal values and healthcare priorities.* They considered both criteria relevant, especially regarding epidemiological data, disease severity and the priorities for which they believe should be pre-determined on a national level by the insured population, i.e. the Slovenian nation.

However, at that point, the debate about medicine availability in Slovenia aroused, encouraged by the following expert’s question:

“*I wonder if we can even talk objectively about taking priorities into account… Is there any medicine that is not available in our country? We have them all…”.*

The expert responsible for reimbursement responded that even if that was almost the case up to date, reimbursing all new medicines even for only a few patients would not be not possible anymore. Two other experts agreed and added that the challenges of medicine reimbursement are becoming greater and that we need to prioritize if we want a sustainable healthcare system.

Furthermore, they expressed that *societal perspective of the disease* and *medicine impact on community* are neglected and missed in the current decision-making process.

*Medicine implementation* was not considered very important but also not negligible.

Moreover, the cardiologist and the public health expert thought that the relevance of each criterion can be adjusted by weighting, as one of them said: “*All aspects need to be considered, also the organizational aspect. But if I had to do it, I would put more weight to some than to others*”.

In the end, one of the clinicians added another point of view regarding reimbursement decision-making criteria:

“*There is another dimension to the criteria which is that they should be known and accepted by the experts as well as by the community”.*

### Themes supporting the structure of criteria

Aside from topics regarding criteria, the experts also discussed general issues in medicine reimbursement, policy making, healthcare legislation, and target populations.

They pointed out some examples of medicine decision-making from other countries, e.g. the United Kingdom where medicine reimbursement decision-making is based mostly on medicines’ cost-effectiveness as a very straight-forward one and too simple to work properly. On the other hand, one pointed out that in the United Kingdom, lay public is included into decision-making, which is also favourable for Slovenia. Additionally, a suggestion how to make decision-making more reasonable was proposed:

“*Is it suitable to talk about a final reimbursement decision? Or should we rather be* re-evaluating *reimbursement* decisions after *some time, e.g. three* years*, and see if a medicine has proven to be as effective in real-life clinical practice as expected.*”

The panel regret that healthcare priority setting is currently based on the interests of particular smaller groups, such as particular patient associations or political groups with power to influence policy-making, which was pointed out at several occasions during the debate by three experts.

Additionally, they discussed defining target population to be reimbursed under social aspects and healthcare priorities. The medicine reimbursement decision-maker explained that target populations should be defined by the public health institutions and further considered in reimbursement decisions within several criteria.

### Priority setting and criteria transferability

The experts’ overall view was that healthcare priorities should be determined based on community consent, regarding the values in society. They believed that prioritizing itself is not a decision-maker’s task, as One expert familiar with health policy-making explained:

“*We exceed the healthcare system here… it is the politics that has determined the priorities,* but *it is supposed to be about the values* in *society”*.

Another expert emphasized that setting priorities should be wisely considered:

“*Priorities should be determined responsibly, and then followed. At the moment, there exists some “positive discrimination”* of *certain diseases,* e.g. *rare diseases...”*

Further, three experts expressed disagreement with some of the priorities on the current list defined within the Health Care and Health Insurance Act, e.g. diabetes, psoriasis, some psychiatrical and neurological diseases, since they believe that there exist conditions much more serious for the patients or such that represent a greater burden than those defined currently.

The experts agreed that some populations should be considered our first concern following the intrinsic human sense of fairness, solidarity and ethics. The public health expert was reflected in the following opinion:

*“I understand the obligations of the healthcare system as following priorities in healthcare which are somehow set by the community… what we, as a community, have decided that our priorities are…”.*

Finally, the experts proposed some ways to help set the priorities. For example:

“*I would set the healthcare priorities based on epidemiological studies – what currently represents the burden or a future projection of what would represent the burden to the community. I would set the priorities based on data.”*

Another example was:

“*If we have a severe disease that affects large population, then this is an absolute national healthcare priority”.*

Lastly, they agreed the decision-making criteria should be unique for medicines, particular priority medicines (e.g. rare diseases) and other health technologies if the set is comprehensive, including all relevant aspects discussed. The regulatory expert with experience in health technology assessment elaborated:

“*If we consider all the categories, the population impact, the burden of disease and ethics – they will all be taken into account for a priority as well… I believe health technologies should be considered holistically – you can apply all aspects either on medicines or on medical devices…”.*

However, they advocated that the use of criteria should be reasonable, allowing for different weights attributed to same criteria in cases of healthcare priority versus non-priority medicines.

## Discussion

The Slovenian healthcare experts see reimbursement decision-making comprehensively: they believe it needs to be fair and based on the community consensus, legally supported healthcare organization, priorities and criteria, and then reasonably performed. Generally, the panel believes that the Slovenian reimbursement decision-making is more all-inclusive than some others (i.e. in the United Kingdom) and therefore more ethical for considering multiple aspects. As in other healthcare systems around the world, the Slovenian healthcare system is also dealing with the problem of affordability of new medicines; however, the general drive in the Slovenian medicine reimbursement is to find a way to fund a new medicine for at least a minimal critical number of patients in need, if there is enough evidence for its efficacy and safety. Therefore, the tendency in medicine reimbursement decision-making is to search for the ways how to allocate the resources to be able to reimburse at least a certain volume of a new medicine.

Still, they exposed the lack of public opinion in decision-making and advocated that common beliefs and values of people financially participating in the healthcare system should represent the basis to determine healthcare priorities, target populations and target medicines to be funded. This may be achieved by considering several criteria and by including lay people in the process or at least assure that the criteria are accepted by them [30]. Also, the panel exposed that there is a huge need to review the national healthcare priorities, despite this being a topic the experts and policy-makers would rather avoid.

Nevertheless, a decision-makers’ list should include disease burden, societal disease perspective and pre-defined national healthcare priorities. It should also comprise the aspects concerning the medicine – its influence on health benefit including quality of life of all concerned, medicine-related costs and willingness to pay and the healthcare setting possibilities for its implementation. Unanimously, all experts believe that none of these criteria could be completely neglected in reimbursement decision-making of whatever health technology. However, they recognized significant differences in criteria relevance.

Medicine’s efficacy and safety seemed the most important aspects to the whole panel to assure health gain where appropriately designed clinical trials are crucial; if they demonstrate efficacy and safety relevant for the clinical practice (using adequate comparators, patient numbers and end-points), they provide strong evidence and make decision-makers’ job easier. Further, re-evaluating past decisions using the new (real-life) clinical data can make decision-making more reasonable.

Further, all experts considered economic aspects the second key criterion where the overall cost and budget impact are two basic elements needed for decision. Also crucial, it is the value the society is willing to attribute to a certain health unit (value of life, value per quality-adjusted life year etc.) and what are the agreed thresholds. The experts complained that these are too weakly established and little known by the public.

Medicine health benefit and economic aspects are fully included in the EUnetHTA and EVIDEM frameworks and also already importantly represented in the existing Slovenian decision-making process [3, 4, 20, 21]. Despite that, the experts suggested some new features: including real-world data, re-evaluating decisions and setting more firm thresholds. Furthermore, societal perspective should be defended when deciding how much to invest.

Disease prevalence and severity representing disease burden closely follow the first two criteria and are recognized worldwide as aspects describing a health problem [20–22]. The experts recognized that effects of disease and medicine occurring at the population level are currently not considered enough. They exposed that we still use an almost completely patient-centred approach while we don’t consider the effects like quality of life of relatives and caregivers (e.g. in evaluating an anti-dementia medicine). They explained that the burden of disease is very important; however, it does not by itself reflect the ‘social aspect’. Therefore, we should take medicine’s capacity to improve community health-related detriments into account in decision-making.

Last but not least, the medicine implementation was at first doubted to be a relevant criterion in decision-making, however, the experts argued this should be considered beside other criteria in order to take healthcare system capacities and ability to use a medicine properly into account and to avoid causing any disparities or difficulties in providing care to the patients.

Finally, the experts agreed that a ‘wise’ use of the criteria needs to be assured in all situations.

Focus panel methodology was chosen to enable the participants to communicate and develop their ideas. The questions originating from the existing criteria could influence the expert’s ideas, however, all experts were well aware of the existing international criteria frameworks before the discussion.

Nevertheless, we derived the proposed criteria set from the opinions of five national healthcare representatives with long-time experience in tackling different healthcare issues. They also spontaneously tended to achieve a common conclusion and had similar motivation for participation: to help improve the decision-making process and access to new medicines. Therefore, their views are a valuable basis for developing renewed criteria for medicines reimbursement decision-making in Slovenia and similar healthcare systems. In further efforts, all aspects should be revealed in even more detail and confirmed by the experts and the Slovenian population.

## Conclusions

The Slovenian healthcare experts advocate considering multiple aspects in decision-making, as proposed by the international frameworks and tools. Medicine health benefit and economic aspects remain the most relevant criteria. The experts suggest re-evaluating decisions with included real-world clinical data and recognize that in the existing process social aspects of disease and medicine impact on the community are largely missed.

## Abbreviations

HIIS: Health Insurance Institute of Slovenia
